# Diagnostic accuracy for the epileptogenic zone detection in focal epilepsy could be higher in FDG-PET/MRI than in FDG-PET/CT

**DOI:** 10.1007/s00330-020-07389-1

**Published:** 2020-10-15

**Authors:** Kazufumi Kikuchi, Osamu Togao, Koji Yamashita, Daichi Momosaka, Tomohiro Nakayama, Yoshiyuki Kitamura, Yoshitomo Kikuchi, Shingo Baba, Koji Sagiyama, Keisuke Ishimatsu, Ryotaro Kamei, Nobutaka Mukae, Koji Iihara, Satoshi O. Suzuki, Toru Iwaki, Akio Hiwatashi

**Affiliations:** 1 Department of Clinical Radiology, Graduate School of Medical Sciences, Kyushu University, 3-1-1 Maidashi, Higashi-ku, Fukuoka, 812-8582 Japan; 2 Department of Molecular Imaging & Diagnosis, Graduate School of Medical Sciences, Kyushu University, 3-1-1 Maidashi, Higashi-ku, Fukuoka, 812-8582 Japan; 3 Department of Neurosurgery, Graduate School of Medical Sciences, Kyushu University, 3-1-1 Maidashi, Higashi-ku, Fukuoka, 812-8582 Japan; 4 Department of Neuropathology Graduate School of Medical Sciences, Kyushu University, 3-1-1 Maidashi, Higashi-ku, Fukuoka, 812-8582 Japan

**Keywords:** Epilepsy, Positron emission tomography, Magnetic resonance imaging, Fluorodeoxyglucose F18, Sensitivity

## Abstract

**Objectives:**

To examine the utility of FDG-PET/MRI in patients with epilepsy by comparing the diagnostic accuracy of PET/MRI and PET/CT in epileptogenic zone (EZ) detection.

**Methods:**

This prospective study included 31 patients (17 males, 14 females) who underwent surgical resection for EZ. All patients were first scanned using FDG-PET/CT followed immediately with FDG-PET/MRI. Two series of PET *plus* standalone MR images were interpreted independently by five board-certified radiologists. A 4-point visual score was used to assess image quality. Sensitivities and visual scores from both PETs and standalone MRI were compared using the McNemar test with Bonferroni correction and Dunn’s multiple comparisons test.

**Results:**

The EZs were confirmed histopathologically via resection as hippocampal sclerosis (*n* = 11, 35.5%), gliosis (*n* = 8, 25.8%), focal cortical dysplasia (*n* = 6, 19.4%), and brain tumours (*n* = 6, 19.4%) including cavernous haemangioma (*n* = 3), dysembryoplastic neuroepithelial tumour (*n* = 1), ganglioglioma (*n* = 1), and polymorphous low-grade neuroepithelial tumour of the young (*n* = 1). The sensitivity of FDG-PET/MRI was significantly higher than that of FDG-PET/CT and standalone MRI (FDG-PET/MRI vs. FDG-PET/CT vs. standalone MRI; 77.4–90.3% vs. 58.1–64.5% vs. 45.2–80.6%, *p* < 0.0001, respectively). The visual scores derived from FDG-PET/MRI were significantly higher than those of FDG-PET/CT, as well as standalone MRI (2.8 ± 1.2 vs. 2.0 ± 1.1 vs. 2.1 ± 1.2, *p* < 0.0001, respectively). Compared to FDG-PET/CT, FDG-PET/MRI increased the visual score (51.9%, increased visual scores of 2 and 3).

**Conclusions:**

The diagnostic accuracy for the EZ detection in focal epilepsy could be higher in FDG-PET/MRI than in FDG-PET/CT.

**Key Points:**

*• Sensitivity of FDG-PET/MRI was significantly higher than that of FDG-PET/CT and standalone MRI (FDG-PET/MRI vs. FDG-PET/CT vs. standalone MRI; 77.4–90.3% vs. 58.1–64.5% vs. 45.2–80.6%, p < 0.0001, respectively).*

*• Visual scores derived from FDG-PET/MRI were significantly higher than those of FDG-PET/CT and standalone MRI (2.8 ± 1.2 vs. 2.0 ± 1.1 vs. 2.1 ± 1.2, p < 0.0001, respectively).*

• *Compared to FDG-PET/CT, FDG-PET/MRI increased the visual score (51.9%, increased visual scores of 2 and 3).*

**Electronic supplementary material:**

The online version of this article (10.1007/s00330-020-07389-1) contains supplementary material, which is available to authorized users.

## Introduction

Epilepsy is a chronic neurological disorder that affects approximately 1% of the global population [1]. In the USA and other industrialised countries, where many antiepileptic drugs are readily available, 30–40% of patients continue to suffer seizures that are not adequately controlled by pharmacotherapy [2]. Treatment of patients with medically intractable seizures accounts for most of the healthcare costs associated with epilepsy [3].

Many patients disabled by epilepsy may be candidates for surgical therapy [4]. Recent advances in diagnostic procedures, particularly neuroimaging, have greatly increased interest in surgical therapy [4]. Resective surgery is a potentially curative therapy, especially in focal epilepsy [5]. Because successful resective surgery depends on the correct localisation of the epileptogenic zone (EZ), preoperative identification is vital [6]. MRI is a powerful tool for identifying lesions causing epilepsy; however, despite recent advances, MRI still fails to reveal any apparent abnormality in approximately 20% of patients with epilepsy [7].

PET has been used to detect abnormalities that may indicate the EZ [5]. For focal cortical dysplasia (FCD), [18F]-fluorodeoxyglucose (FDG)-PET is known to be more sensitive than MRI [8]. A previous study revealed that PET could show beneficial information in patients with MRI/electroencephalogram-negative epilepsy [6]. However, the detection rate of EZs in epilepsy using FDG-PET or FDG-PET/CT has been reported to be only 36–73% [6]. Thus, it remains difficult to detect EZs on FDG-PET/CT due to the lack of soft-tissue contrast [9]. This shortcoming mandates an additional, separate MRI exam. Furthermore, ionising radiation is inherently inevitable with PET/CT systems, and this is also a significant shortcoming, especially in children [9].

Hybrid PET/MRI systems have the potential to combine the superior soft-tissue contrast of MRI and the metabolic characterisation of FDG-PET in a single exam. This may be an ideal imaging tool for EZ detection because it can evaluate both anatomical and functional information simultaneously. Hybrid PET/MRI systems have already been applied in cancer research, such as in brain, head and neck, lung, musculoskeletal, and pancreatic cancers [10]. To the best of our knowledge, there are no data directly comparing the diagnostic accuracy of FDG-PET/CT and FDG-PET/MRI in patients with histopathologically confirmed epilepsy as a reference standard. We hypothesised that the diagnostic accuracy of the EZ detection in focal epilepsy may be higher in FDG-PET/MRI than in FDG-PET/CT. Thus, the purpose of this study was to examine the utility of FDG-PET/MRI in patients with epilepsy by comparing the diagnostic accuracy of PET/MRI and PET/CT in EZ detection.

## Materials and methods

This single-institute prospective study was approved by the institutional review board. Informed consent was obtained from all patients.

### Patients

From November 2014 to March 2018, 86 consecutive patients with focal epilepsy were enrolled in this study. Inclusion criteria were as follows: (1) an International League Against Epilepsy (ILAE) outcome scale [11] score of 5 or less and (2) availability of both FDG-PET/CT and FDG-PET/MRI results. The exclusion criteria were as follows: (1) unknown EZ (*n* = 24), (2) no surgery (*n* = 26), and (3) surgery without resection, such as multiple subpial transection, corpus callosotomy, and vagus nerve stimulation (*n* = 5). As a result, a total of 31 patients (17 males, 14 females; median age, 31 years, age range, 8–58 years) were enrolled.

### PET/CT system

A biograph mCT PET/CT system (Siemens Healthcare) was used according to a standard clinical protocol [12]. Patients were instructed to have no caloric intake for 4 h prior to FDG administration. All patients were normoglycaemic (blood glucose below 150 mg/dL) at the time of FDG injection. After the injection of a weight-based dose of FDG (4.0 MBq/kg), patients rested for 60 min in a dark quiet room to allow for tissue uptake. PET scans were performed for 10 min using 3D acquisition and time-of-flight technology. The crystal of the PET/CT system was lutetium oxyorthosilicate, and the imaging matrix was 400 × 400. Each dataset consisted of 90 transaxial PET images with a 2-mm slice thickness and a 256-mm field-of-view (voxel: 2 × 2 × 2 mm^3^). CT for attenuation correction was acquired for each patient with the same protocol (120 kVp, 50–135 mAs, detector 64 rows × 1.2 mm). Images were reconstructed using a time-of-flight and commercially available technique, the TrueX (Siemens) technique, with an all-pass filter (iteration, 8; subsets, 21).

### PET/MRI system

All patients were scanned using PET/MRI within 30 min after the PET/CT exam using the Ingenuity TF PET/MRI system (Philips Healthcare). After the scout image was taken and a 3D-T1-weighted image was acquired to correct attenuation [10], patients underwent PET imaging with 3D-ordered subset expectation maximisation (3D-OSEM) and time-of-flight. The field-of-view for the PET imaging was 576 × 576 mm^2^ (recon. voxel: 2 × 2 × 2 mm^3^). After PET imaging, 3D fluid-attenuated inversion-recovery (FLAIR) images were obtained using a turbo-spin echo sequence with repetition time/inversion time/echo time: 4800/1650/293 ms; flip angle, 180°; turbo-spin echo factor, 182; bandwidth, 1187.1 Hz; field-of-view, 250 × 250 × 220 mm^3^; matrix, 252 × 250 × 220; thickness of image, 1 mm; number of excitations, 1; number of slices, 220; and spectral inversion-recovery was applied for fat suppression; SENSE factor, 2. The scan time in our protocol was approximately 60 min.

### Observer testing

Five board-certified radiologists namely one PET/MRI specialist, one nuclear medicine specialist, and three neuro-specialists (22, 21, 20, 13, and 7 years of experience, respectively) who were blinded to patient information conducted the observer tests. Each observer attended two reading sessions held at least 1 month apart to minimise learning effects [13]. They read either FDG-PET/CT or FDG-PET/MRI in the first session and vice versa in the second session. After the second session, we performed an additional standalone MRI session to verify how FDG-PET affected the diagnostic performance. All images of all 31 patients were presented in a randomised order every session. In the standalone MRI session, both conventional and dedicated-epilepsy MR protocols were used, which included axial and paracoronal FLAIR images reconstructed in the long axis and perpendicular to the hippocampi.

### Sensitivity

Firstly, the observers were instructed to observe the laterality of FDG uptake using axial as well as coronal PET images. Secondly, the observers determined the anatomical parts of the EZ using all three types of images, i.e., PET, CT, and MRI, as well as fused PET/MRI or PET/CT. EZ detection was confirmed when both the laterality and the anatomical part of the EZ were detected. The sensitivity of each system was calculated from the EZ detection results from both PET systems plus standalone MRI. The operative report was used as a reference standard when readers disagreed.

### Quantitative visual assessment

The observers also evaluated the boundary of the lesion using a 4-point visual score. We used the modified Paldino et al method [9]. The various scores were defined as follows:

- Score 4: clear detection of EZ in terms of both laterality and border
- Score 3: clear detection of EZ laterality, but a border that was a little obscure
- Score 2: possible detection of EZ laterality and an unclear border
- Score 1: poor quality for detection of EZ laterality and border

The observers were able to easily adjust the window level and window width, as well as the degree of transparency using the workstation (SYNAPSE VINCENT; Fujifilm Medical).

### Statistical analysis

The sensitivity of FDG-PET/MRI, FDG-PET/CT, and standalone MRI for the EZ detection was compared using the McNemar test with Bonferroni correction. The intraclass correlation coefficient (ICC) was calculated and interpreted as follows: excellent agreement, ICC > 0.8; good agreement, ICC > 0.6; moderate agreement, ICC > 0.4; and poor agreement, ICC ≤ 0.4, as proposed earlier among the five observers [14]. The 4-point visual score was also compared for the three systems using Dunn’s multiple comparisons test. All statistical analyses were performed using a commercial software program (GraphPad Prism 7.0; GraphPad Software). A *p* value < 0.05 was considered statistically significant.

## Results

### Distributions of the epileptogenic zone and pathologies

Based on the inclusion and exclusion criteria, 31 patients (17 males, 14 females) were enrolled. The EZs were confirmed histopathologically via resection as hippocampal sclerosis (HS; *n* = 11, 11/31 = 35.5%), gliosis (gliosis; *n* = 8, 8/31 = 25.8%), FCD; *n* = 6, 6/31 = 19.4%), and brain tumours (*n* = 6, 6/31 = 19.4%) including cavernous haemangioma (*n* = 3), dysembryoplastic neuroepithelial tumour (*n* = 1), ganglioglioma (*n* = 1), and polymorphous low-grade neuroepithelial tumour of the young (PLNTY) (*n* = 1). There were 33 EZs in the 31 patients with epilepsy (two patients had multifocal EZs, e.g., parietotemporal and frontoparietal lobes). Table 1 summarises the distributions of EZs and pathologies.

**Table 1:** Distributions of epileptogenic zone and the associated pathologies

Location	Subjects	Right	Left
Frontal lobe	6	3 [3 gliosis]	3 [2 FCD Ib, 1 FCD IIb]
Parietal lobe	1	0	1 [1 FCD IIa]
Temporal lobe	22	11 [4 HS I, 3 gliosis, 2 CH, 1 FCD IIb, 1 PLNTY]	11 [5 HS I, 2 HS II, 1 CH, 1 DNT, 1 FCD Ib, 1 GG]
Occipital lobe	0	0	0
Multifocal	2	1 [1 gliosis (parietotemporal)]	1 [1 gliosis (frontoparietal)]

*Abbreviations*: *CH*, cavernous haemangioma; *DNT*, dysembryoplastic neuroepithelial tumour; *FCD*, focal cortical dysplasia; *GG*, ganglioglioma; *HS*, hippocampal sclerosis; *PLNTY*, polymorphous low-grade neuroepithelial tumour of the young

### Observer testing

**Fig. 1:**
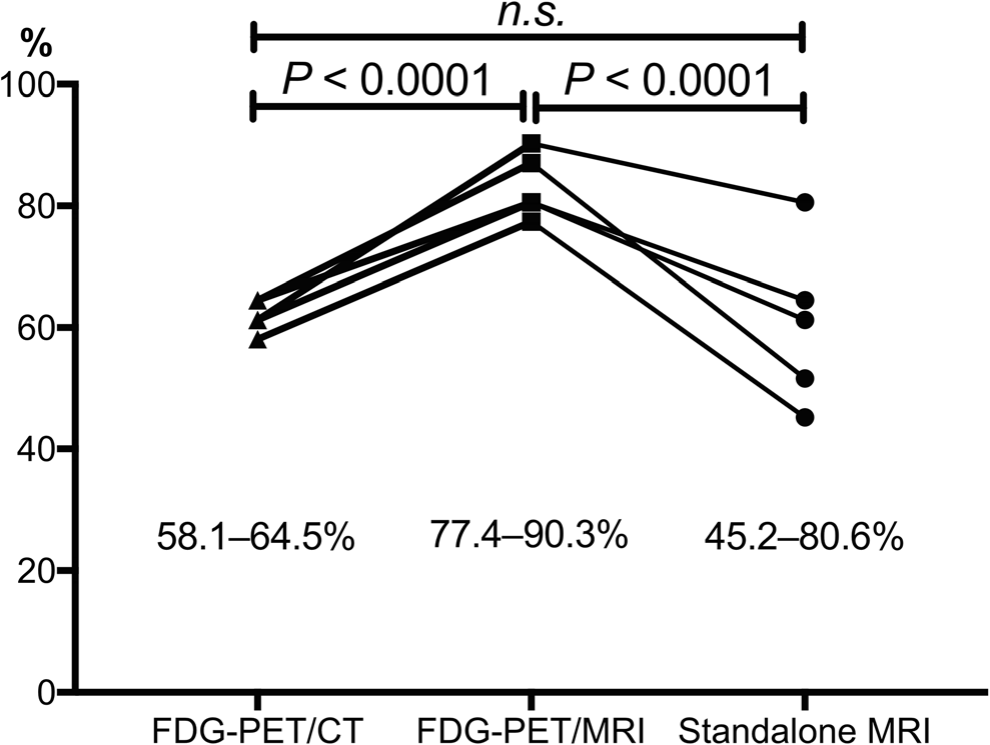
Sensitivity of epileptogenic zone detection for each [18F]-fluorodeoxyglucose (FDG)-PET system. The diagnostic sensitivity of PET/MRI is significantly higher than that of PET/CT (*p* < 0.0001). *FDG*, fluorodeoxyglucose

**Fig. 2:**
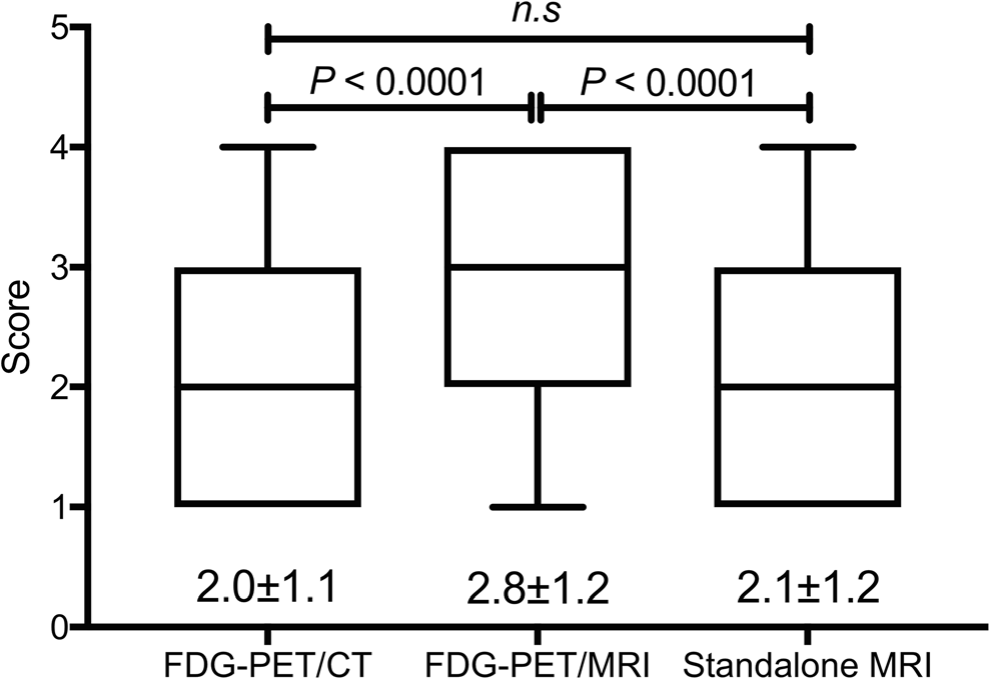
The 4-point visual score of the epileptogenic zone boundary for each [18F]-fluorodeoxyglucose (FDG)-PET system. The visual score of PET/MRI is significantly higher than that of PET/CT (*p* < 0.0001). Notably, the visual score of PET/MRI is nearly equal to 3, which suggests that it is useful for determining the surgical margin. *FDG*, fluorodeoxyglucose

ICCs among the five observers of FDG-PET/MRI and FDG-PET/CT were 0.45 and 0.42, respectively. Observations made by the five board-certified radiologists showed that the sensitivity derived from FDG-PET/MRI was significantly higher than that from FDG-PET/CT and standalone MRI (Fig. 1, Table 2; FDG-PET/MRI vs. FDG-PET/CT vs. standalone MRI; 77.4–90.3% vs. 58.1–64.5% vs. 45.2–80.6%, *p* < 0.0001, respectively). The visual scores derived from FDG-PET/MRI were significantly higher than those of FDG-PET/CT, as well as standalone MRI (Fig. 2, Table 3; 2.8 ± 1.2 vs. 2.0 ± 1.1 vs. 2.1 ± 1.2, *p* < 0.0001, respectively). Compared to FDG-PET/CT, FDG-PET/MRI increased the visual score (51.9%, increased visual scores of 2 and 3, Table 3), which suggested that the anatomical contrast improvement with MRI had an impact on the readers, especially in terms of determining the borders between normal and abnormal regions. These results demonstrated the superiority of FDG-PET/MRI compared to FDG-PET/CT for the detection of the EZ in patients with focal epilepsy and that FDG-PET enhanced the sensitivity and visuality with standalone MRI.

**Table 2:** Detection of epileptogenic zones with FDG-PET/MRI vs. FDG-PET/CT *and FDG-PET/MRI vs. standalone MRI*

31 patients × 5 readers		FDG-PET/MRI	
		+	–
FDG-PET/CT	+	86	6
	–	37	26
		*p* value^a^	< 0.0001
31 patients × 5 readers		FDG-PET/MRI	
		+	–
Standalone MRI	+	90	4
	–	35	26
		*p* value^a^	< 0.0001

*Abbreviations*: *FDG*, fluorodeoxyglucose F18

^a^Calculated by the McNemar test

**Table 3:** Improvement of visual score between FDG-PET/MRI vs. FDG-PET/CT and FDG-PET/MRI vs. standalone MRI

FDG-PET/MRI	Increase of visual score			*p* value^a^
31 patients × 5 readers	1	2	3	
vs. FDG-PET/CT (52.3%, 81/155)	48.1% (39/81)	34.6% (28/81)	17.3% (14/81)	< 0.0001
vs. standalone MRI (49.0%, 76/155)	61.8% (47/76)	32.9% (25/76)	5.3% (4/71)	< 0.0001

*Abbreviations*: *FDG*, fluorodeoxyglucose F18

^a^Calculated by Dunn’s multiple comparison test

**Fig. 3:**
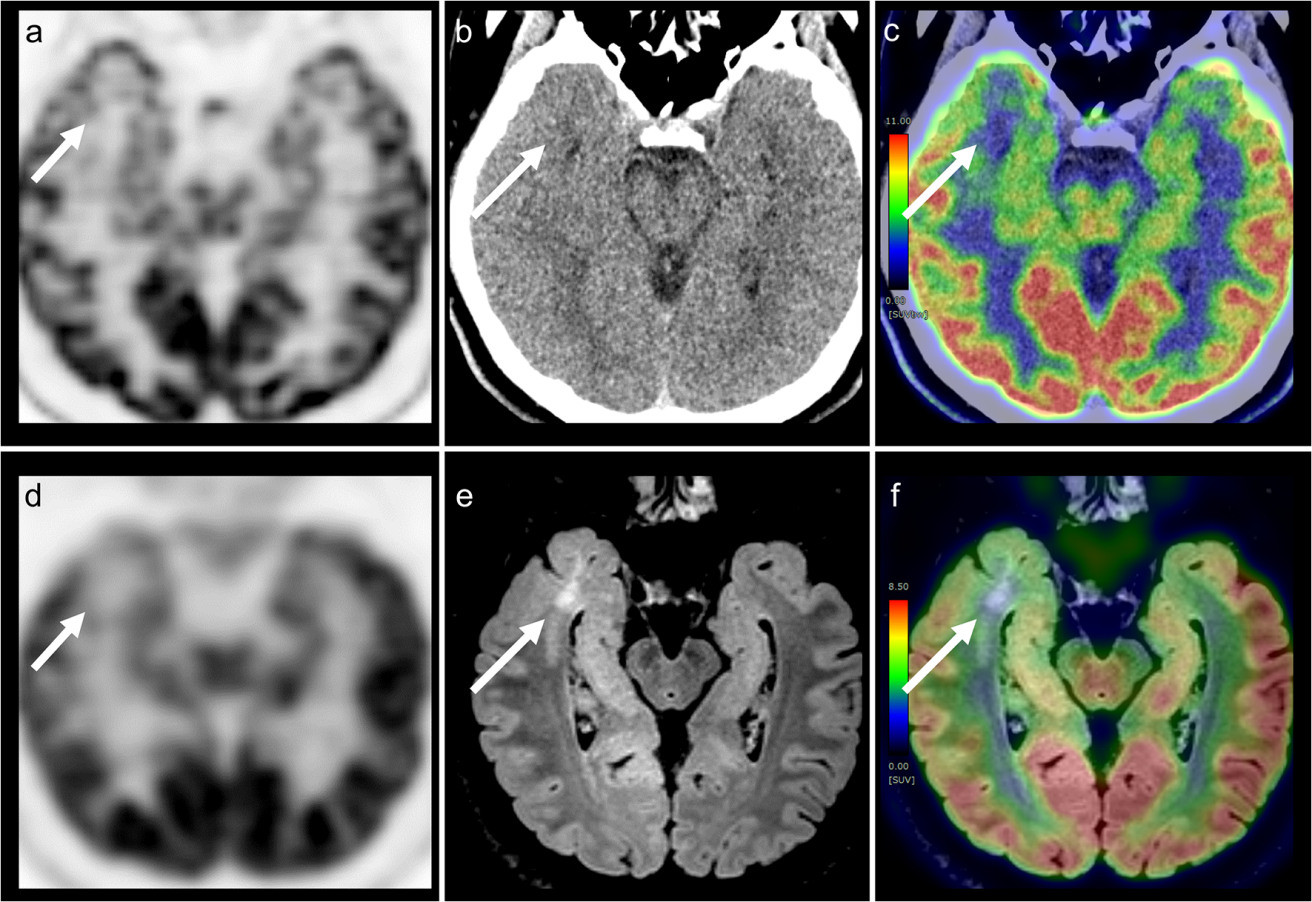
Dedicated PET from PET/CT (**a**) and PET/MRI (**d**), CT (**b**), FLAIR (**e**), PET/CT (**c**), and PET/MRI (**f**) acquired with [18F]-fluorodeoxyglucose (FDG) examinations of the brain in an 18-year-old male with a polymorphous low-grade neuroepithelial tumour of the young (PLNTY). A focal area of decreased tracer accumulation is shown in the right temporal lobe, which is consistent with the epileptogenic zone (arrows). After surgery, this patient was completely seizure free (International League Against Epilepsy; ILAE outcome scale 1). *FDG*, fluorodeoxyglucose; *FLAIR*, fluid-attenuated inversion-recovery

**Fig. 4:**
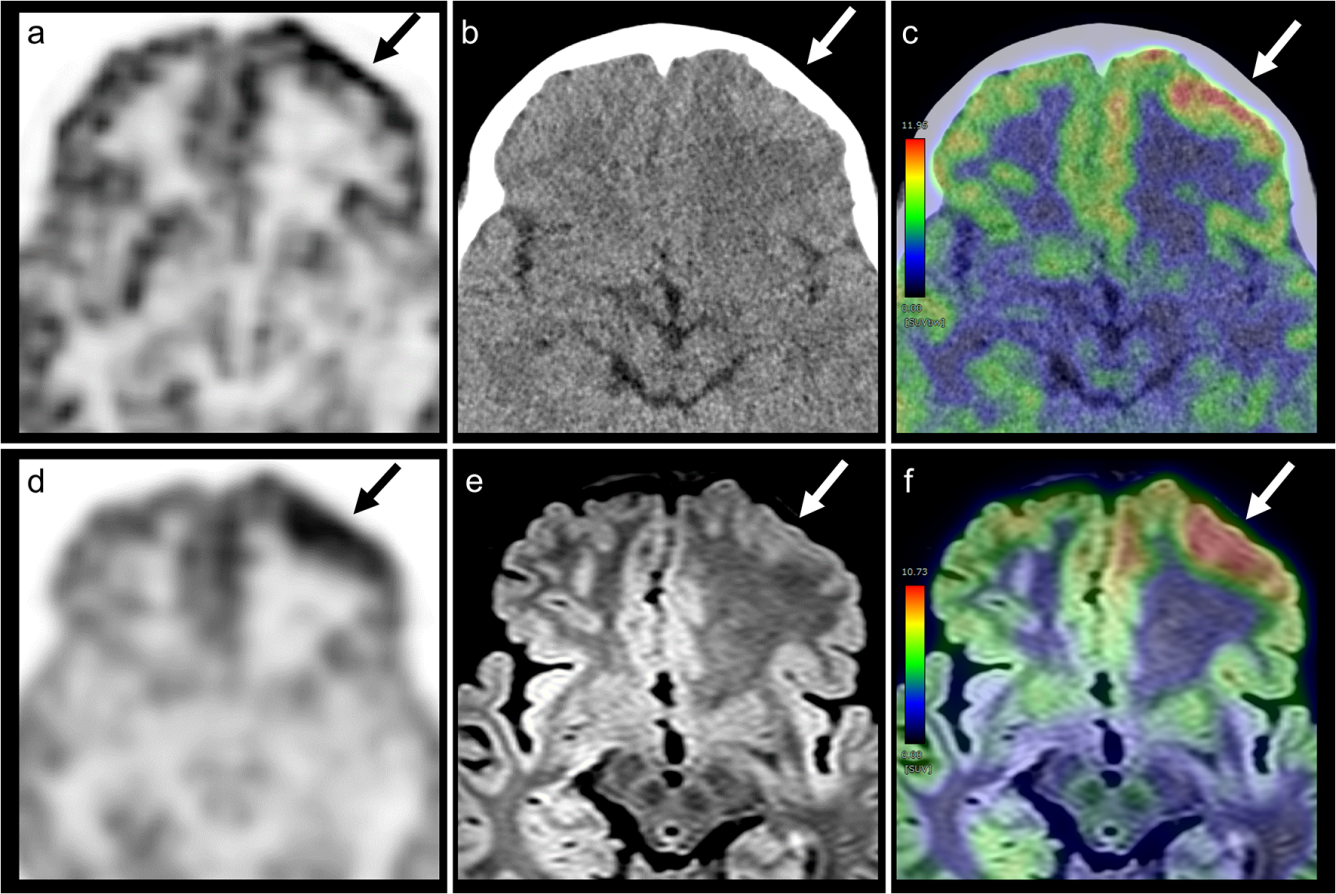
Dedicated PET from PET/CT (**a**) and PET/MRI (**d**), CT (**b**), FLAIR (**e**), PET/CT (**c**), and PET/MRI (**f**) acquired with [18F]-fluorodeoxyglucose (FDG) examinations of the brain in an 8-year-old male with focal cortical dysplasia (FCD), type Ib. A focal area of increased tracer accumulation is shown in the left frontal lobe immediately after ictus, which is consistent with a seizure focus (arrows). Notably, the abnormality of cortical thickness is more clearly depicted on PET/MRI than on PET/CT. After surgery, this patient was completely seizure free (International League Against Epilepsy; ILAE outcome scale 1). *FDG*, fluorodeoxyglucose*; FLAIR*, fluid-attenuated inversion-recovery

Figures 3 and 4 show representative cases with PLNTY and FCD type Ib, respectively.

## Discussion

This study demonstrated that FDG-PET/MRI showed higher sensitivity and higher visual scores than FDG-PET/CT on the observer test. These results suggest that the diagnostic accuracy of FDG-PET/MRI was superior to that of PET/CT for the detection of the EZ in patients with focal epilepsy. Our findings are consistent with those of previous studies showing that FDG-PET/MRI was superior or not inferior to PET/CT for the diagnosis of epilepsy in children [9]. To our knowledge, there are very little data that directly evaluates and compares the diagnostic accuracy of PET/MRI and PET/CT in patients with focal epilepsy that has been confirmed histopathologically through surgical resection. Furthermore, we demonstrated that the PET/MRI visual score was nearly equal to 3, suggesting that it is useful for determining the surgical margin. Compared to standalone MRI, FDG-PET/MRI could enhance both sensitivity and visuality of EZ detection.

HS was the largest cause of an EZ, followed by gliosis, then FCD and brain tumours in our study cohort. HS was the most common cause of temporal lobe epilepsy, and this finding was consistent with previous reports [15]. Although the histopathologically specific cause was often unknown, even after resection [16], there were still therapeutic effects because the patients in this present study had an ILAE scale after resection of 5 or less.

The site of abnormal FDG uptake may be connected to the EZ, and this finding is useful for surgical planning and the placement of the intracranial electroencephalogram. Even though FDG abnormalities are found, the lesion border is still indistinct on CT, and in contrast, MRI has superior tissue contrast and is useful for determining the surgical margin.

In the observer testing, ICCs among the five observers were moderate. The differences among observers may be due to their speciality. Neuroradiologists’ scores did not differ much between FDG-PET/MRI and standalone MRI compared to those of other specialists. We found that FDG-PET/MRI showed higher sensitivity compared to FDG-PET/CT despite the FDG decay of PET/MRI in our observer test with moderate ICC. The sensitivities of FDG-PET/CT and FDG-PET/MRI in the current study, at 61.3–64.5% and 77.4–90.3%, respectively, were similar to previously reported sensitivities of 60–90% and 78–100%, respectively [6, 9].

A previous study of fused PET/MRI, which is acquired with MRI and PET separately and processed after acquisition, reported that coregistration is helpful because the colour-coded technique seemed to identify subtle decreases in FDG hypometabolism [17]. However, the non-synchronised and non-simultaneous acquisition of different functional parameters might lead to potential biases in EZ localisation [6]. Thus, hybrid PET/MRI has advantages because it can provide simultaneous acquisition of both anatomical and functional information in the same pathophysiological state.

We also found that FDG-PET/MRI showed a higher visual score compared to FDG-PET/CT and standalone MRI in our observer test. Despite recent advances in image fusion software, it is still difficult to retrospectively generate well-matched fusion images using MRI and PET images acquired separately with different machines [10]. Furthermore, temporal differences are crucial in epilepsy because the FDG uptake with inter-ictal and post-ictal states differs dramatically. As observed in Figs. 3 and 4, our cases showed opposite FDG uptakes because of the different ictal state. FDG-PET/MRI can be considered clinically useful in improving visibility using isovoxel thin-sliced FLAIR images in addition to temporal and spatial synchronisation. The observer test results of our study support the concept that FDG-PET/MRI can enhance the non-invasive detection of patients with focal epilepsy because the visual score of PET/MRI was nearly equal to 3, which indicates clear detection of the EZ laterality, as well as an almost well-defined border of the lesion.

This study had some limitations. First, there was some patient selection bias. Because the reference standard was based on histopathology in our study, no resection cases were excluded. This may have led to an overestimation of the stated sensitivity by excluding negative and inconclusive objects. Second, visual assessment is a subjective method. Objective methods, such as statistical parametric mapping analysis and quantitative PET including commercial databases of healthy controls [18, 19], should be used in future studies. Third, PET reconstruction is better for PET/CT than for PET/MRI. This is due to the decay of FDG and difference in scanner technology. This has a small impact on clinical usage, however, as clinically all three types of images are typically read concurrently rather than PET alone. Fourth, standardised uptake values (SUVs) on the FDG-PET/MRI system could be underestimated compared to FDG-PET/CT. [18F]-FDG static SUV measurements were performed with the segmentation algorithm for attenuation correction in the PET/MRI system, which cannot separate cortical bone from soft tissue [10]. The segmentation algorithm is a simple method that is widely used for attenuation correction on PET/MRI. A recent study revealed that an atlas-based algorithm may improve this underestimation [18]. However, the main purpose of this study was to detect the EZ qualitatively, and thus, the difference in SUV values had less effect on the results. Some EZs in this study were not detected on either PET/CT or PET/MRI, and other modalities, such as electroencephalogram and magnetoencephalography, are also used to make a final determination in our institution. It is important to integrate the findings of these modalities with clinical information.

In conclusion, the diagnostic accuracy for the EZ detection in focal epilepsy could be higher in FDG-PET/MRI than in FDG-PET/CT.

## Electronic supplementary material

Supplementary Figure 1 Study flow diagram *FDG*, fluorodeoxyglucose (DOCX 190 kb)

## Abbreviations

EZ: Epileptogenic zone
FCD: Focal cortical dysplasia
FLAIR: Fluid-attenuated inversion-recovery
HS: Hippocampal sclerosis
ICC: Intraclass correlation coefficient
ILAE: International League Against Epilepsy
PLNTY: Polymorphous low-grade neuroepithelial tumour of the young
