# Supplementary figures and images for: Diagnostic accuracy for the epileptogenic zone detection in focal epilepsy could be higher in FDG-PET/MRI than in FDG-PET/CT
Source: Eur Radiol. 2020 Oct 15;31(5):2915–22. doi: 10.1007/s00330-020-07389-1 (PMC8043950; doi:10.1007/s00330-020-07389-1)

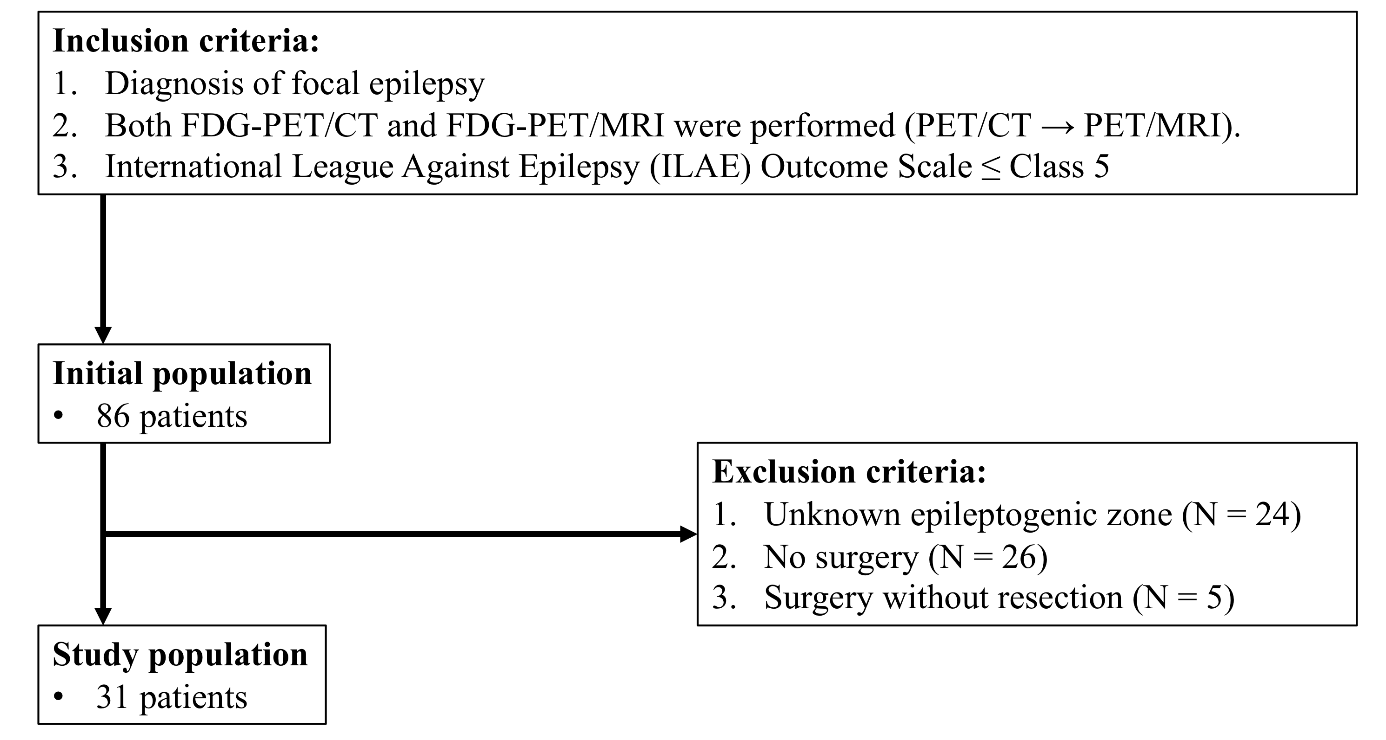


**Supplementary Figure 1:** Study flow diagram

*FDG*, fluorodeoxyglucose

Supplement: Supplementary file 1 — Study flow diagram FDG, fluorodeoxyglucose (DOCX 190 kb) [file 330_2020_7389_MOESM1_ESM.docx]
